# Elemental composition of primary lamellar bone differs between parous and nulliparous rhesus macaque females

**DOI:** 10.1371/journal.pone.0276866

**Published:** 2022-11-01

**Authors:** Paola Cerrito, Bin Hu, Justin Z. Goldstein, Rachel Kalisher, Shara E. Bailey, Timothy G. Bromage

**Affiliations:** 1 Department of Anthropology, New York University, New York, New York, United States of America; 2 New York Consortium in Evolutionary Primatology, New York, New York, United States of America; 3 Department of Molecular Pathobiology, New York University College of Dentistry, New York, New York, United States of America; 4 Collegium Helveticum, ETH, Zürich, Switzerland; 5 Department of Anthropology, Texas State University, San Marcos, Texas, United States of America; 6 Joukowsky Institute for Archaeology and the Ancient World, Brown University, Providence, Rhode Island, United States of America; Universitair Kinderziekenhuis Koningin Fabiola: Hopital Universitaire des Enfants Reine Fabiola, BELGIUM

## Abstract

Extracting life history information from mineralized hard tissues of extant and extinct species is an ongoing challenge in evolutionary and conservation studies. Primary lamellar bone is a mineralized tissue with multidien periodicity that begins deposition prenatally and continues until adulthood albeit with concurrent resorption, thus maintaining a record spanning several years of an individual’s life. Here, we use field-emission scanning electron microscopy and energy-dispersive X-ray analysis to measure the relative concentrations of calcium, phosphorous, oxygen, magnesium and sodium in the femora of seven rhesus macaque with known medical and life-history information. We find that the concentration of these elements distinguishes parous from nulliparous females; that in females calcium and phosphorus are lower in bone formed during reproductive events; and that significant differences in relative magnesium concentration correlate with breastfeeding in infants.

## Introduction

The reconstruction of demographic patterns and life-history scheduling of both extant and extinct mammalian species is an ongoing challenge in several disciplines, including evolutionary biology, conservation studies and biological anthropology. The reconstruction of life-history scheduling has been routinely addressed via the histological analysis of mineralized tissues. These tissues act as recoding structures [1], keeping track of the physiological changes experienced by an organism in the form of accentuated growth lines and changes in elemental composition. A large body of work spanning several decades has shown that a variety of both intrinsic and extrinsic events, including birth [2, 3], reproduction [4], weaning [5], menopause [6], illnesses [7], dietary changes [8] and climate variation [9–11], leave a permanent record in calcified tissues which is preserved even in fossil specimens [12]. The microstructural organization of teeth is not altered during life [13]. As such, teeth have been the structure of choice for the identification and chronological reconstruction of past physiological changes in mammals. Fortunately, primary lamellar bone is not significantly remodeled in shorter-lived species such a rhesus macaques [14] and other catarrhine primates [15]. Large segments of this tissue are also maintained well into adulthood in humans [9], with some variation across geographic groups.

Lamellar bone is an incremental tissue formed according to a coordinated internal biological rhythm. It is the same rhythm visible in the Retzius Periodicity (RP) of enamel [16] and it remains constant throughout the entire life of the individual. Its recording sensitivity is high. In rhesus macaques one lamella has a thickness in the order of 3.5–4μm and a period of four days (rarely five, as per personal observation of TGB). While its period of registration spans prenatal life to adulthood, the duration of the record is limited due to resorption of earlier-formed bone.

The skeleton participates to whole organism physiology and reflects alterations in endogenous and exogenous conditions by undergoing changes in both growth rate [9] and elemental composition [17]. Research on humans [18] and rodents [19] has shown that several adaptations such as increased absorption, decreased renal excretion and increased skeletal resorption allow for the calcium (Ca) and phosphorous (P) needs of the forming fetuses to be met without necessitating a concurrent increase in maternal Ca and P intake. Especially during the last term of gestation, maternal serum Ca and P, but not magnesium (Mg) [17] or sodium (Na) [20] are decreased. This is remarkable considering that, in rodents, up to ~80% of the Ca present in maternal circulation is transferred, each hour, to the developing litter [17].

Elemental analysis employing laser ablation inductively coupled plasma mass spectrometry on skeletal remains associated with known ages at life-history milestones has shown that weaning is recorded in the teeth of walruses [5], humans [21] and macaques [22]. Similarly, Changes in zinc concentrations in teeth have been reported to reflect the onset of sexual maturity in walruses [23], whereas declining fertility has been shown to correlate with increased copper concentrations in rat teeth [24]. Other research on both primates and cetaceans, have employed scanning electron microscopy (SEM) equipped with energy dispersive X-ray spectroscopy (EDS) to assess the changes in major and minor element concentrations over time [25, 26]. However, the chemical signature of reproductive events in hard tissues is yet to be characterized in any mammalian species.

In this study we use field emission (FE) SEM-EDS to measure changes in five major and minor element concentrations (oxygen, calcium, magnesium, sodium and phosphorus) in the femora of seven male (n = 3) and female (n = 4) rhesus macaques with known medical and life-history information. Given the participation of the skeleton to the metabolic demands of reproduction, we predict the following:

1. The elemental composition across all bone of parous females will be significantly different from that of males and nulliparous females.
2. P and Ca concentrations will be lower in the tissues formed during pregnancy and lactation.
3. Maternal relative percent concentrations of Mg will be higher in the bone formed during pregnancy and lactation since other elements, such as P and Ca, will be transferred to a proportionally greater extent than Mg.
4. Relative percent concentrations of Mg in infants will be lower in the tissues formed during breastfeed than after weaning is completed, since other elements, such as P and Ca, will be transferred to a proportionally greater extent than Mg.

## Materials and methods

### Specimen information

The present work did not require IACUC approval as it neither used live subjects, nor did it require sacrificing any animals. All the individuals in this study died from causes unrelated to this research. The research adhered to the American Society of Primatologists’ Principles for the Ethical Treatment of Nonhuman Primates. The skeletonized specimens were acquired from the Caribbean Primate Research Center (CPRC). Prior to their death the animals were housed at the Sabana Seca field station of the University of Puerto Rico where they were provisioned with monkey chow. Veterinarians at the Field Station monitored and recorded information on their health and reproductive history. The IDs of the seven femora used in this study, together with each individuals’ sex, parity and dates of birth and death are reported in Table 1. The medical and life history information is reported in S1 Table. In order to calculate the growth rate of the lamellar bone [16], we also acquired the value of the RP from the individual dental sections of the same individuals [27]. This value (four days) was the same for all seven specimens.

**Table 1:** Specimen information.

Specimen ID	DOB	DOD	Sex	Parity
AV27	18-Jan-07	18-Nov-12	M	
MA024	30-Apr-10	21-Nov-11	F	0
CK85	6-Mar-16	13-Feb-18	M	
BD24	21-Jul-08	17-Apr-13	F	0
M361	11-Jul-94	1-Oct-11	M	
M526	24-Jul-98	20-Sep-16	F	9
M591	21-May-00	15-Aug-16	F	1

For each individual: date of birth (DOB) and death (DOD), sex and parity (for females).

### Block preparation and imaging

We selected the right femora of each of the seven individuals. We removed a ~1.0 cm block from the midshaft of each femur. Each block was cleaned, dehydrated using progressively higher-concentration solutions of ethanol, and then embedded in poly methyl methacrylate (PMMA) following procedures described in detail by Goldman and colleagues [28]. Both sides of the block were then ground to 1200 grit finish using progressively graded carbide papers and polished with a 0.1-μm diamond suspension. The blocks were mounted on an EXAKT (Oklahoma City, OK, USA) plastic slide using cyanoacrylate adhesive.

Each block was imaged in reflected circularly polarized light (CPL) using a Leica DMRXE microscope fitted with an automated X, Y and Z stage. We collected a montaged micrograph of each block at a resolution of 1.3 μm / pixel. Given the high resolution with which these micrographs were acquired, the identification of contiguous patches of lamellae was unambiguous. On each block, we used the reflected CPL montages to identify two regions fulfilling our criteria of contiguity across which we collected the data, from here onwards identified as Transect 1 and Transect 2. The CPL montages were used as references for the SEM imaging, which was performed before proceeding with the EDS data collection. Once we identified on the EDS the same field of view that we had chosen with the CPL images, we verified that the patches of lamellae were indeed contiguous, and only then proceeded to acquire EDS data.

### Elemental data collection

To be suitable for high-vacuum SEM imaging and EDS analysis, we uniformly carbon-coated the blocks using a Denton Desk V (Denton Vacuum, Moorestown, NJ, USA). The thickness of the carbon coating is ~20nm. It is also worth noting that Monte Carlo simulations [29, 30] clearly demonstrate that the depth of penetration of the electron beam means that signal emanates from a thickness of bone that is about 2 orders of magnitude deeper than the carbon coating thickness.

All data were collected using a Bruker Quantax 200 XFlash 6160 EDS detector (Bruker, Billerica, MA, USA) coupled with a Zeiss Gemini-300 FE-SEM (Carl Zeiss Microscopy, White Plains, NY, USA). Before collecting the data, we confirmed by imaging in the SEM that the surface was free of any topographical relief. We acquired the data with the following SEM settings: in high vacuum mode, with an accelerating voltage of 15kV, with an electron beam aperture of 60μm, in high current mode and with a working distance of 8.5mm, which is the analytical working distance of this particular microscope and detector setup. We acquired the data in line scan mode and collected one line scan per segment. The spot size was set each time to match the radius of the interaction volume of the beam, which was consistently ~1.0μm since all the samples had similar composition and were analyzed with the same experimental conditions. The scan dwell time was set at 64μs, and the spectrometer throughput at 275Kcps. To ensure that we had at least 10,000 counts for each peak, the scan time was manually determined for each specimen since they varied in dimension (length of the segment). We used the zero kV peak to make sure that each spectrum was properly aligned. After acquisition was complete, we used the P/B-ZAF standardless analysis quantification model [31] in interactive mode which allowed us to manually validate each peak.

Each transect (two per specimen, 14 in total) was acquired in several separate segments (56 in total), moving from the periosteal surface towards the endosteal one. The spectra corresponding to each segment is available online as supplementary data (see the Data Availability Statement). Each segment was chosen to avoid osteocyte lacunae and dehydration cracks as best we could. Details regarding the segments collected for each transect are available in Table 2. The elements that were detected with sufficient counts per peak in significantly above-background concentrations in all 56 segments were: C, O, P, Ca, Na, Mg. We saved our data as.csv files in which for each point across the femoral transect we recorded the number of counts for each of the elements detected in above-threshold concentrations.

**Table 2:** Dimensional information regarding each segment and transect for all seven femora.

Transect (number of segments comprising it)	Total length of the transect (μm)	Average growth rate (μm/year)	Corresponding years of bone	Estimated age at periosteal margin	Estimated age at endosteal margin
**AV27_trans1 (segments 1,2,3,4,5)**	1439	379	3.797	5.900	2.103
**AV27_trans2 (segments 6,7,8,9)**	1159	279.8	4.142	5.900	1.758
**MA024_trans1 (segments 1,2,3)**	1025	539.2	1.901	1.600	-0.301
**MA024_trans2 (segments 4,5)**	533	310.4	1.717	1.600	-0.117
**CK85_trans1 (segments 1,2,3)**	957	499.8	1.915	1.900	-0.015
**CK85_trans2 (segments 4,5)**	476	337	1.412	1.900	0.488
**BD24_trans1 (segments 1,2,3,4,5)**	1188	360.5	3.295	4.750	1.455
**BD24_trans2 (segments 6,7,8)**	906	283.1	3.200	4.750	1.550
**M361_trans1 (segments 1,2,3,4,5)**	1817	367.1	4.950	10.849	5.900
**M361_trans2 (segments 6,7,8,9,10,11)**	2206	360.4	6.121	12.020	5.900
**M526_trans1 (segments 1,2,3,4,5)**	1256	307.2	4.089	8.839	4.750
**M526_trans2 (segments 6,7,8,9)**	973	294.9	3.299	8.839	5.539
**M591_trans1 (segments 1,2,3,4)**	1618	276.2	5.858	7.458	1.600
**M591_trans2 (segments 6,7,8,9)**	1510	282.8	5.339	6.939	1.600

For each transect: number of segments comprising the transect, total length of the transect (in micrometers), average growth rate (in micrometers per year), corresponding years of bone, chronological age (in years) estimated at the periosteal and endosteal margin of the transect. The information for each transect used to derive the growth rates is available in S2 Table.

### Thin section preparation and imaging

The carbon-coated side of each block was mounted on an EXAKT (Oklahoma City, OK, USA) plastic slide using cyanoacrylate adhesive. Using a Buehler (Lake Bluff, IL, USA) Isomet 1000 saw we sectioned the block so that a ~150-μm thick section remained attached to the slide. Each section was then ground to 100 ± 10 μm and polished with a 0.1-μm diamond suspension. The resulting thin sections were imaged using the same Leica DMRXE microscope but in transmitted circularly polarized light (CPL). We acquired a montaged image of each section at a resolution of 0.325 μm / pixel. Since we mounted the carbon-coated surface directly on the plastic slide, without polishing or otherwise touching the surface, these micrographs clearly show the segments that were targeted by the electron beam of the SEM.

### Chronological age assignation

**Fig 1:**
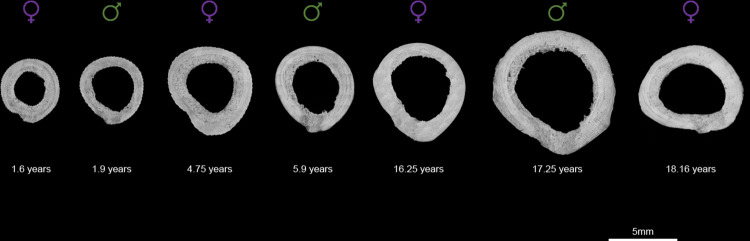
Montages of micrographs acquired in reflected light of the blocks of the seven femora. For each femur we report the sex and age of each. The micrographs are oriented with the posterior portion towards the bottom and the medial one towards the left.

To analyze the possible correlations between the elemental data and the differing physiological states of the animals we assigned a chronological age range to each transect (Table 2). First, we used the montages acquired in CPL mode to measure transect-specific growth rates of the bone. Along each transect of each individual we measured the thickness of the lamellae five times and calculated the average value (S2 Table). By knowing the RP values (four days), we were able to infer the transect-specific average yearly growth rate. For the four younger individuals (MA024, CK85, AV27 and BD24) the femoral diameter was still increasing at the time of death. Therefore, we aged the segments of primary lamellar bone from the periosteal margin towards the endosteal one, assuming that the age at the periosteal one corresponded to the age at death. For the older individuals M361, M526 and M591 primary lamellar growth had ceased before their death. We aged the segments of these individuals from the endosteal margin of the primary lamellar bone towards the periosteal one and derived the age of the endosteal one from corresponding diameters of the immature individuals (Fig 1 and S1–S7 Figs). The only exception is Transect 2 of M526 for which, due to extensive remodeling and cortical drift it was not possible to match the endosteal margin with corresponding primary bone of younger female individuals. Hence, this transect was aged from the periosteal towards the endosteal region by matching its periosteal margin to that of Transect 1 of the same individual. Primary lamellar bone growth ceased between seven and eight years for females, and up to 12 years for males, likely relating to their increase in body mass well into adulthood [32, 33].

### Data preparation

First, we discarded the data points collected over any cracks. Then, within each data file and corresponding to each acquisition point across the femoral transect, we added the following variables: age; category and specific type of event relating to reproduction and weaning; type of individual (male, nulliparous female, parous female).

Illnesses and traumas were detailed in the life histories, so we were able to track in time the progression of an affliction. For gestation, we began at the point in bone corresponding to an offspring’s date of birth and counted back 5.5 months (c. 165 days) prior [34–37]. Lactation periods may vary by individual, so for its coding we began at the point in bone corresponding with an offspring’s date of birth and counted ahead 5 months, as this was found to be the minimum duration of breastfeeding in various studies [34, 38, 39]. Ages at weaning were available for 4/7 macaques, and thus this period was averaged (9.5 months) and applied to the two individuals for which data was lacking but which experienced breastfeeding during the periods of cementum formation that we analyzed. The averaged period of 9.5 months falls in line with previous scholarship on macaque lactation duration [38]. All cementum deposited before the age at weaning was considered “suckling”. We use suckling to indicate the period from birth to first introduction of solid foods and weaning as the period between the first introduction of solid foods and the transition to al 100% non-milk diet.

In most cases, there was only one Specific Event for a given period, but for some individuals, two Specific Events may have been experienced simultaneously. These cases were mostly related to females who experienced illness while pregnant or lactating. In these scenarios, the reproductive specific event took precedent and thus we coded it accordingly.

Carbon coating our specimens artificially increased concentrations of C, rendering absolute elemental values problematic. Instead, we analyzed the data in a way that they would reveal any changes in elemental composition over time (and physiological events). To this end, we pre-processed our original data in two different ways, each one aimed at addressing a different question:

1. Within each data file we scaled the data for each element, which was originally recorded as net total (NETTO) counts, such that the minimum value would be 0 and the maximum would be 1. This type of processing provides information regarding the behavior of a single element over time. We use this data to address prediction 2. This dataset is referred to as “normalized data”.
2. Within each sample, we eliminated the values for C and then calculated the percent concentration for each element at each acquisition point. For example, at t_1_%Mg = NETTO_Mg_ / (NETTO_Mg_ + NETTO_Na_ + NETTO_P_ + NETTO_Ca_ + NETTO_O_)*100. This implies that the resulting relative percent concentrations reveal how changes in the concentration of one element are correlated with changes in the concentration of the other ones. This type of data allows us to analyze of the simultaneous behavior of several elements as their numerical relationship is maintained, even if all the values are artificially increased by the absence of C. We used these data to address predictions 1, 3 and 4. This dataset is referred to as “percentage data”.

### Data analysis

All data analysis was carried out using R version 3.6.3 (R Core Team, 2020). For prediction 1, we used proprietary scripts (S1 File, functions *pair*.*test*.*all*, *remove*.*ns* and *final*) to identify individuals who were significantly different from all other individuals belonging to different groups, defined as either male, nulliparous or parous female. Additionally, we performed a principal component analysis (PCA) to visually appreciate the separation between the different types of individuals. For predictions 2, 3 and 4 we ran Bonferroni-corrected two-tailed pairwise t-tests to assess whether there were significant differences in elemental composition associated with either maternal reproductive events or infant breastfeeding. Primary lamellar bone stopped forming before M591 experienced her single reproductive event. Since the age range of primary lamellar bone for M591 (5.3 to 7.5 years) did not encompass the age at which this female gave birth (7.8 years), data from this individual was not included in the analyses for predictions 2 and 3, which test the elemental composition of bone formed during reproductive activity.

## Results

### Prediction 1

**Fig 2:**
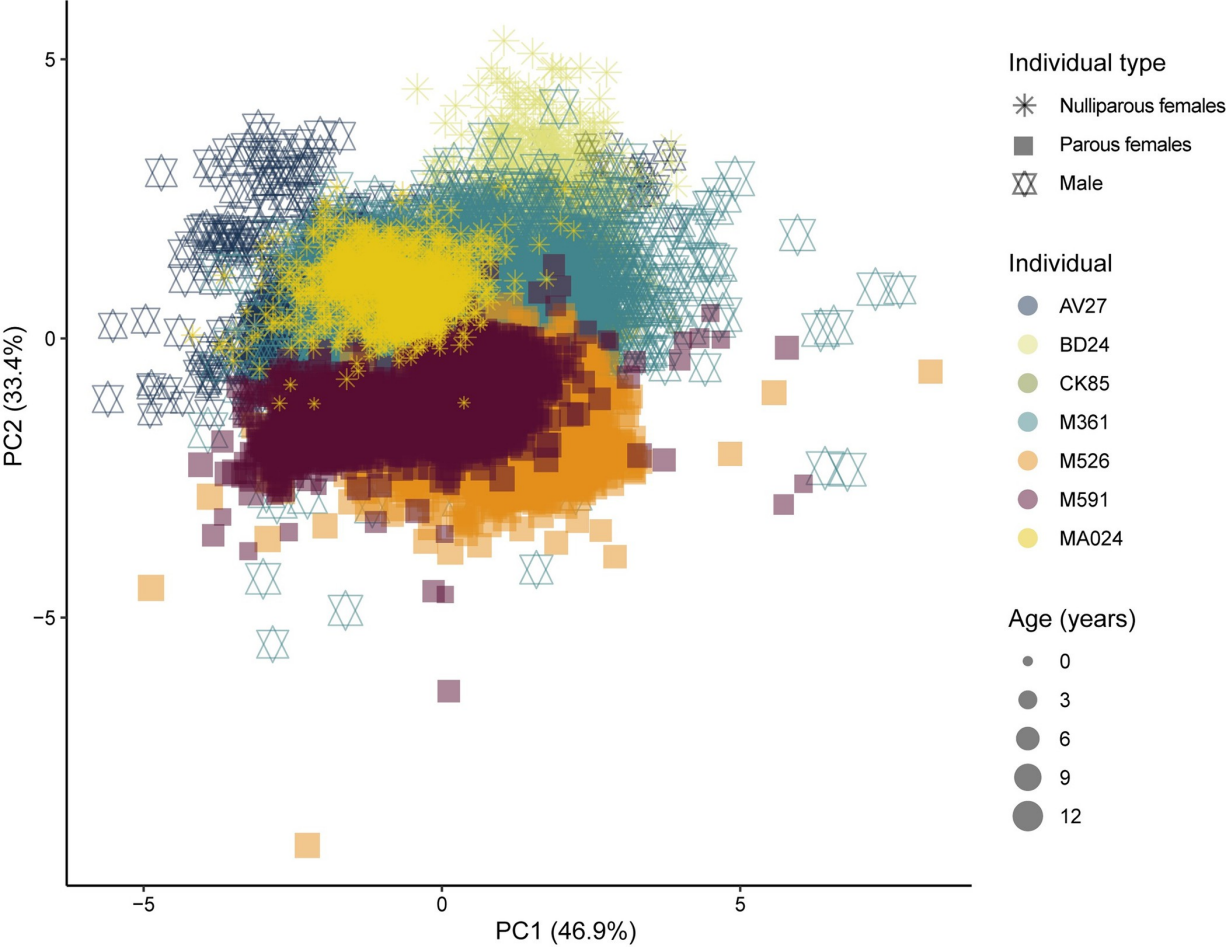
PCA on percentage data grouped by individual. Both segments from the same individual are pooled together. M526 and M591 are the only parous females, while the other adult female in the dataset (BD24) is nulliparous.

Our results support the prediction that the elemental composition of reproductive females is different from that of nulliparous females and young and old males. The average elemental percentage of Ca, O, P, Mg and Na, across both segments is significantly different (p < 0.05) for the two parous females (M591 and M526, S3 Table). This is readily appreciable in the PCA (Fig 2), where there is almost no overlap between nulliparous and parous females. We also conducted a post-hoc PCA to assess whether this difference is a function of the age of each data point but found that the difference between parous females and other individuals is present regardless of the specific chronological age of data points (S8 Fig).

### Prediction 2

**Fig 3:**
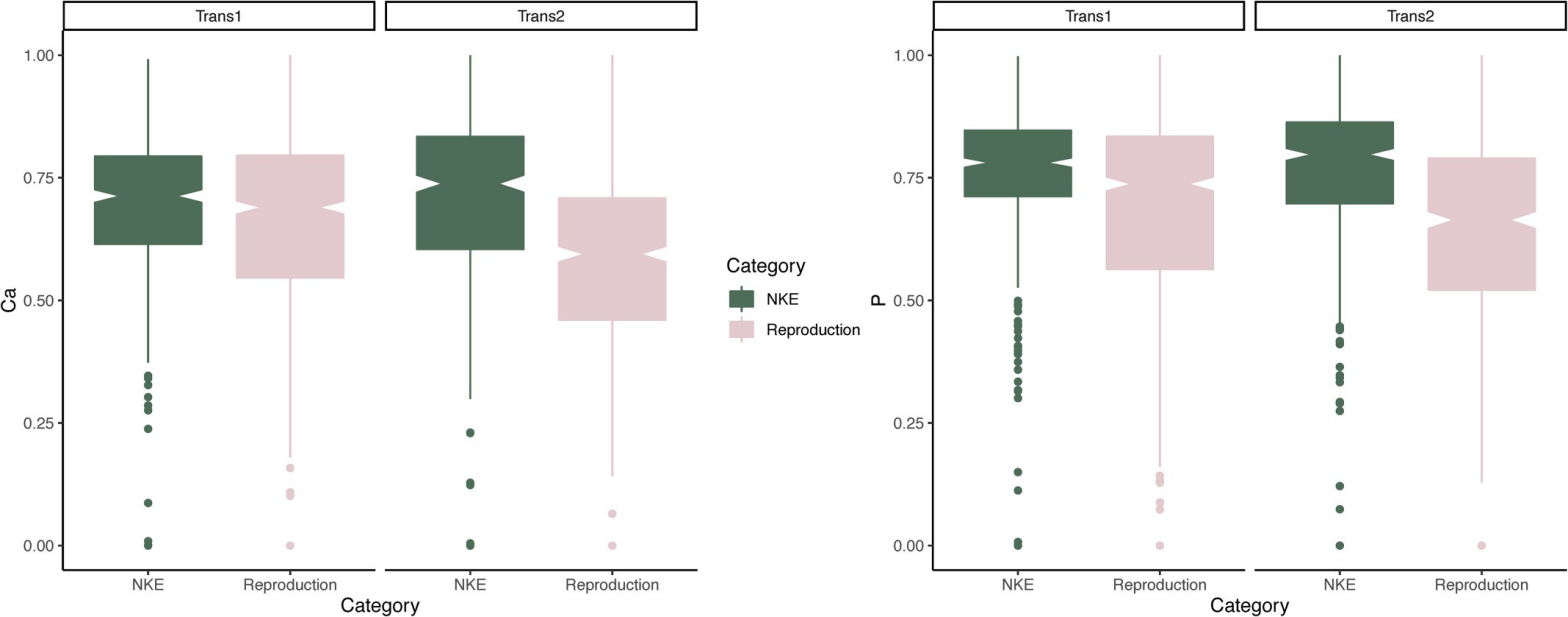
Boxplot showing the difference in normalized concentrations of calcium (left) and phosphorous (right) in the two transects (Trans1 and Trans2) of the bone of M526 formed during reproductive events (gestation and lactation) or during times of no known physiologically challenging events (NKE). Calcium and phosphorus concentrations during reproductive events are significantly lower (p<0.05) in both transects.

Our results fully support our prediction (Fig 3). Normalized Ca values are lower during reproductive events in Transect 1 (p = 0.025) and Transect 2 (p < 0.00001) of M526. Normalized P values are also lower during reproductive events in both Transect 1 and Transect 2 (p < 0.00001) of M526. Both Ca and P normalized values are significantly lower, in M526, during lactation than during gestation in Transect 2 (p < 0.00001), but not in Transect 1, where values are instead lower during gestation (p < 0.00001) (S10 and S11 Figs).

### Prediction 3

We do not find conclusive support for prediction 3 (S12 Fig), as Mg relative percent concentration is lower in the bone formed during reproductive events only in Transect 2 (p < 0.00001), but not in Transect 1 (p = 0.88) of M526.

### Prediction 4

**Fig 4:**
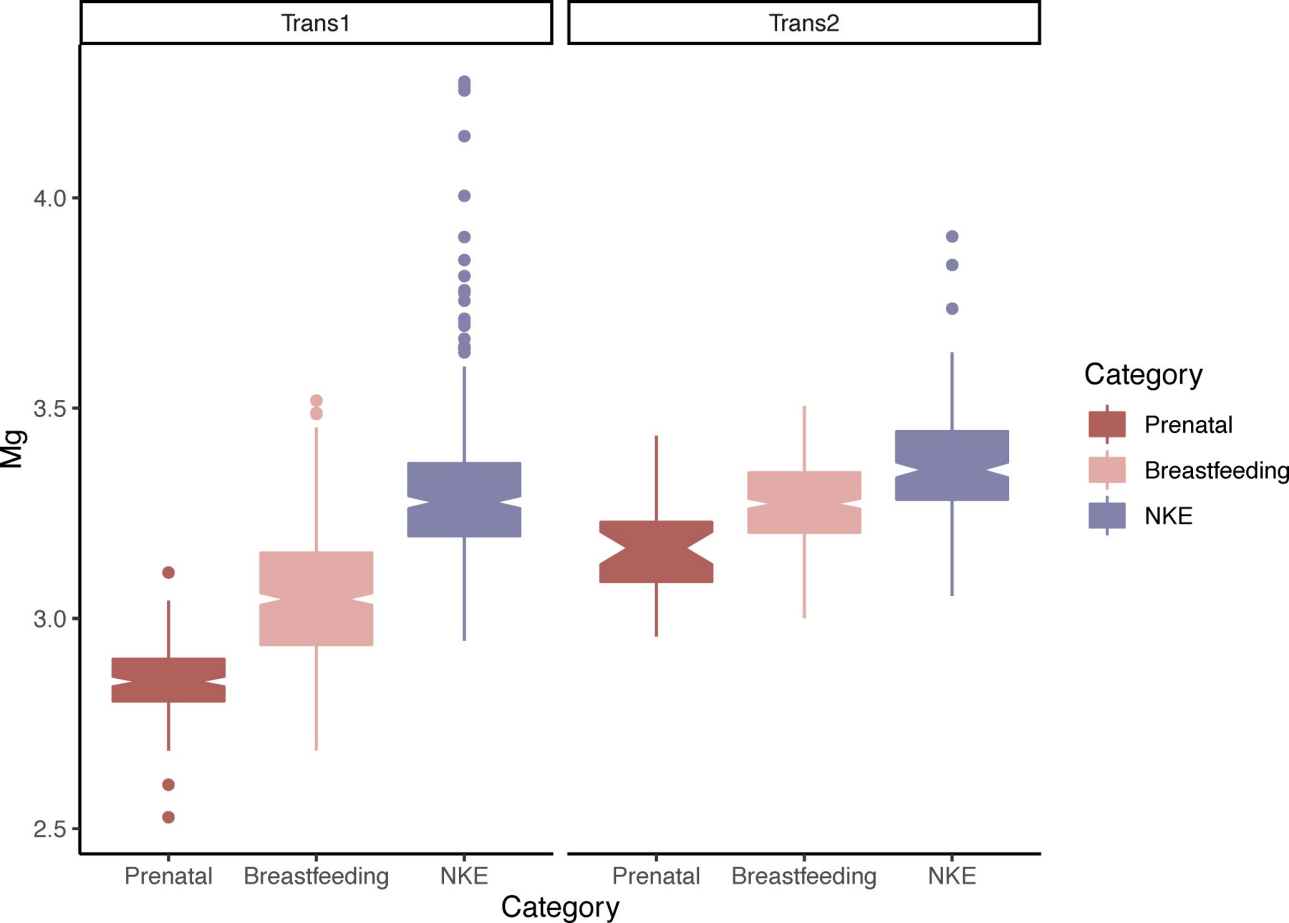
Boxplot showing the difference in relative percent concentrations of magnesium in the bone of MA024 formed prenatally, during breastfeeding (suckling and weaning) and during times of no known physiologically challenging events (NKE). The difference is significant in both transects (p<0.00001).

Prediction 4 is fully supported by our results. Mg relative percent concentration is significantly lower during breastfeeding (p < 0.00001) in both transects of MA024 (Fig 4) and CK85 (S13 Fig). We find also that Mg relative percent concentration is significantly lower in prenatal cortical bone than in the postnatal cortical bone formed both during and after breastfeeding. This is true in both transects of MA024, which is the only individual for which prenatal bone is preserved (Fig 4).

## Discussion

As per prediction 1, we find that there is a significant difference (S3 Table) in relative percent concentration of the five elements considered, between parous females and all other individuals, regardless of the age at which the bone was formed. It is important to notice that one of the parous females included in our sample (M591) had not yet experienced her single reproductive event during bone formation. Therefore, all the bone sampled for this female was formed while she was still nulliparous. One could therefore hypothesize that the difference detected between parous and nulliparous females is instead reflecting a difference between younger and older females, but this is not the case. The observed difference between parous and nulliparous females is independent of age (S8 and S9 Figs). Our results are in agreement with the recent work [40] showing that stable isotopes record sex-specific, but not age-specific changes in rhesus macaques. Work investigating the effects of parity on bone mineral content and density in rhesus macaques has also found that bone characteristics differ between age-matched parous and nulliparous females [41]. These combined findings indicate a retroactive effect of reproduction on bone elemental composition, as previously deposited bone likely contributes to meeting the elemental demands of the developing fetuses [42]. It is also possible that our records fail to report any unsuccessful pregnancy that M591 might have experienced before the first one reported at 7.8 years. Indeed, the mean age at first birth in rhesus macaques is 3.8 years [43], which is four years before the first reproductive event recorded for M591.

In agreement with the vast body of literature documenting the physiological response to the mineral needs of the developing fetus and infant [17–19, 44], bone formed during gestation and lactation has lower concentrations of both Ca and P.

The combined findings of prediction 1 and prediction 2 indicate that: overall Ca concentrations are lower in parous females compared to other individuals, and that they are even significantly lower in the bone of these females formed during reproductive events as bone resorption increases [45]. Conversely, we found no changes in the concentrations of O (S14 Fig).

In agreement with prediction 3 and partially with prediction 4, Mg relative percent concentrations are significantly different in the maternal bone formed during gestation and lactation, and in the bone formed while an infant is breastfeeding. Since the data pre-processing that we performed returns relative percent concentration of each element at each acquisition point across the femoral transects, our results indicate that during reproduction Mg might be lost by the mother proportionally less than the other elements, while concurrently received proportionally less than other elements by the breastfeeding infant. This is in agreement with research on humans showing that serum Mg is minimally altered during reproductive activity [46–48], while serum Ca and P decrease during the same time period [44]. Furthermore, in MA024, for which the endosteal-most portion of the transects is of prenatal formation, we observe an even lower concentration of Mg than during the breastfeeding (suckling and weaning) phases (Fig 4). This suggests a delay in Mg transfer from the maternal organism until after the birth of the fetus. This delay in Mg transfer has also been directly observed in the body fluids of dam and fetal rabbits [49].

Due to the margin of error likely associated with the chronological age assigned to each bone transect, we did not test for elemental signatures of more punctual events, such as physical trauma.

The findings regarding the elemental changes associated with reproduction are of relevance as the detection of parturitions from mineralized tissues is still a vastly unexplored area of research with significant implications for evolutionary, conservation and archaeological studies. However, given the small size of our sample and limited taxonomic breadth, further research on more animals, preferably from wild populations, is necessary to determine the scalability of these results. It would be important to use femora from wild populations as the present specimens were both provisioned and sheltered from extreme climatic events. It is therefore possible that the signal of reproductive events and weaning that we detected could be masked, in wild populations, by physiological responses to changing diets and environments. From an analytical perspective, studies on animals with lower lamellar growth rate would benefit from an increase in the temporal resolution of the data, while those on species with higher growth rate (e.g., humans) would likely have to employ sampling methods with higher spatial resolution (e.g. laser-ablation inductively coupled mass-spectrometry). Indeed, in our sample rhesus macaque primary lamellar bone growth rate is the range of 0.75 to 1.25 μm/day, while in humans a range of 0.63 to 0.88 μm/day has been reported [50]. Hence, at a given spatial resolution (e.g., 1μm, as in the present study) the temporal resolution for macaques is slightly higher than for humans. Finally, the present work has the caveat of not providing absolute quantitative values of elemental concentrations that can be associated with either reproduction or suckling. Our results are relative either to other animals (e.g., parous females vs. nulliparous ones), or to different periods of a same animal’s life (e.g. reproductive events vs. no physiological stressors) and therefore need reference data to be interpreted. Future work employing absolute quantification methods (such as laser ablation inductively coupled mass spectrometry) are necessary in order to provide absolute quantitative values corresponding to specific physiological events. Quantitative data, especially of elements present in low concentrations, can also be successfully collected at below micrometer spatial resolution using wavelength dispersive spectroscopy (WDX) [51]. EDX technique has the advantage of being much faster and allowing whole spectrum acquisition, however it does have high detection limits, which make it not suitable for trace element identification. Conversely, WDX is much more time consuming as the entire range of wavelengths must be scanned one at the time, but this allows for much lower detection limits. An additional advantage of WDX comparing to EDX is that it is suitable also for the quantification of irregularly shaped surfaces [52]. It therefore appears to be a trade-off between the two techniques, with EDX being more suitable for exploratory analysis of a wide range of elements, and WDX best suited for hypothesis-driven experiments targeting a small number of elements. The present work should therefore serve as foundational to future research employing different analytical methods to target elements present in low concentrations.

## Supporting information

S1 Fig Montaged micrograph of the femur of MA024 obtained in reflected light. The red lines indicate the locations of transects 1 and 2. For each transect we report the length in micrometers; the yearly growth rate; the age range covered by the transect. For a description of the method used to derive the ages, see the Methods section of the main manuscript.
(TIF)

S2 Fig Montaged micrograph of the femur of M591 obtained in reflected light. The red lines indicate the locations of transects 1 and 2. For each transect we report the length in micrometers; the yearly growth rate; the age range covered by the transect. For a description of the method used to derive the ages, see the Methods section of the main manuscript.
(TIF)

S3 Fig Montaged micrograph of the femur of M526 obtained in reflected light. Montaged micrograph of the femur of M526 obtained in reflected light. The red lines indicate the locations of transects 1 and 2. For each transect we report the length in micrometers; the yearly growth rate; the age range covered by the transect. For a description of the method used to derive the ages, see the Methods section of the main manuscript.
(TIF)

S4 Fig Montaged micrograph of the femur of M361 obtained in reflected light. The red lines indicate the locations of transects 1 and 2. For each transect we report the length in micrometers; the yearly growth rate; the age range covered by the transect. For a description of the method used to derive the ages, see the Methods section of the main manuscript.
(TIF)

S5 Fig Montaged micrograph of the femur of CK85 obtained in reflected light. The red lines indicate the locations of transects 1 and 2. For each transect we report the length in micrometers; the yearly growth rate; the age range covered by the transect. For a description of the method used to derive the ages, see the Methods section of the main manuscript.
(TIF)

S6 Fig Montaged micrograph of the femur of BD24 obtained in reflected light. The red lines indicate the locations of transects 1 and 2. For each transect we report the length in micrometers; the yearly growth rate; the age range covered by the transect. For a description of the method used to derive the ages, see the Methods section of the main manuscript.
(TIF)

S7 Fig The red lines indicate the locations of transects 1 and 2. For each transect we report the length in micrometers; the yearly growth rate; the age range covered by the transect. For a description of the method used to derive the ages, see the Methods section of the main manuscript.
(TIF)

S8 Fig PCA of relative percentage data grouped by individual type (both segments from the same individual are pooled together) and colored according to the age associated with each datapoint of each individual. As visible by the superimposition between blue and yellow datapoints, age is not a discriminating factor.
(TIF)

S9 Fig PCA of relative percentage data of only females, colored by parity state (blue = nulliparous, yellow = parous). The data from both segments of the same individual are pooled together. There is clear separation between parous and nulliparous females. Dot size is proportional to age. It is clearly visible that the separation between parous and nulliparous females is independent of age (dot size).
(TIF)

S10 Fig Boxplot showing the difference in relative percent concentrations of calcium in the bone of M526 formed during gestation and during lactation. Ca values are significantly lower only in Transect 2 (p<0.00001).
(TIF)

S11 Fig Boxplot showing the difference in relative percent concentrations of phosphorous in the bone of M526 formed during gestation and during lactation. P values are significantly lower only in Transect 2 (p<0.00001).
(TIF)

S12 Fig Boxplot showing the difference in relative percent concentrations of magnesium in the bone of M526 formed during reproduction (gestation and lactation) or during times of no known physiologically challenging events (NKE). There is a significant difference only in Transect 2 (p<0.001).
(TIF)

S13 Fig Boxplot showing the difference in relative percent concentrations of magnesium in the bone of CK85 formed during breastfeeding (suckling and weaning) or during times of no known physiologically challenging events (NKE). The difference is significant in both transect 1 and transect 2 (p<0.00001).
(TIF)

S14 Fig Boxplot showing the difference in relative percent concentrations of oxygen in the bone of M526 formed during reproduction (gestation and lactation) or during times of no known physiologically challenging events (NKE). (TIF)

S1 Table List of age at known impactful physiological events for each individual. Ages are reported in years. The duration of each medical event was inferred from the duration of the medical treatment received by the animal, while the duration of gestation was assumed to be the same for all individuals and taken from the published literature, as reported in the Supplementary Methods section.
(XLSX)

S2 Table Detailed measurements for each transect. For each transect: five measures of lamella thickness and corresponding yearly growth rate; average (avg) value of lamella thickness and corresponding growth rate, together with the standard deviation (sd).
(XLSX)

S3 Table Results of the raw (p) and Bonferroni-corrected (p.adj) pairwise t-tests between individuals of different types (males, parous and nulliparous females). Values reported as 0 are actually extremely small, but below the reporting threshold of the pairwise.t.test function in R.
(XLSX)

S1 File Supplementary methods and scripts. (DOCX)
