# Supplementary figures and images for: Elemental composition of primary lamellar bone differs between parous and nulliparous rhesus macaque females
Source: PLoS One. 2022 Nov 1;17(11):e0276866. doi: 10.1371/journal.pone.0276866 (PMC9624403; doi:10.1371/journal.pone.0276866)

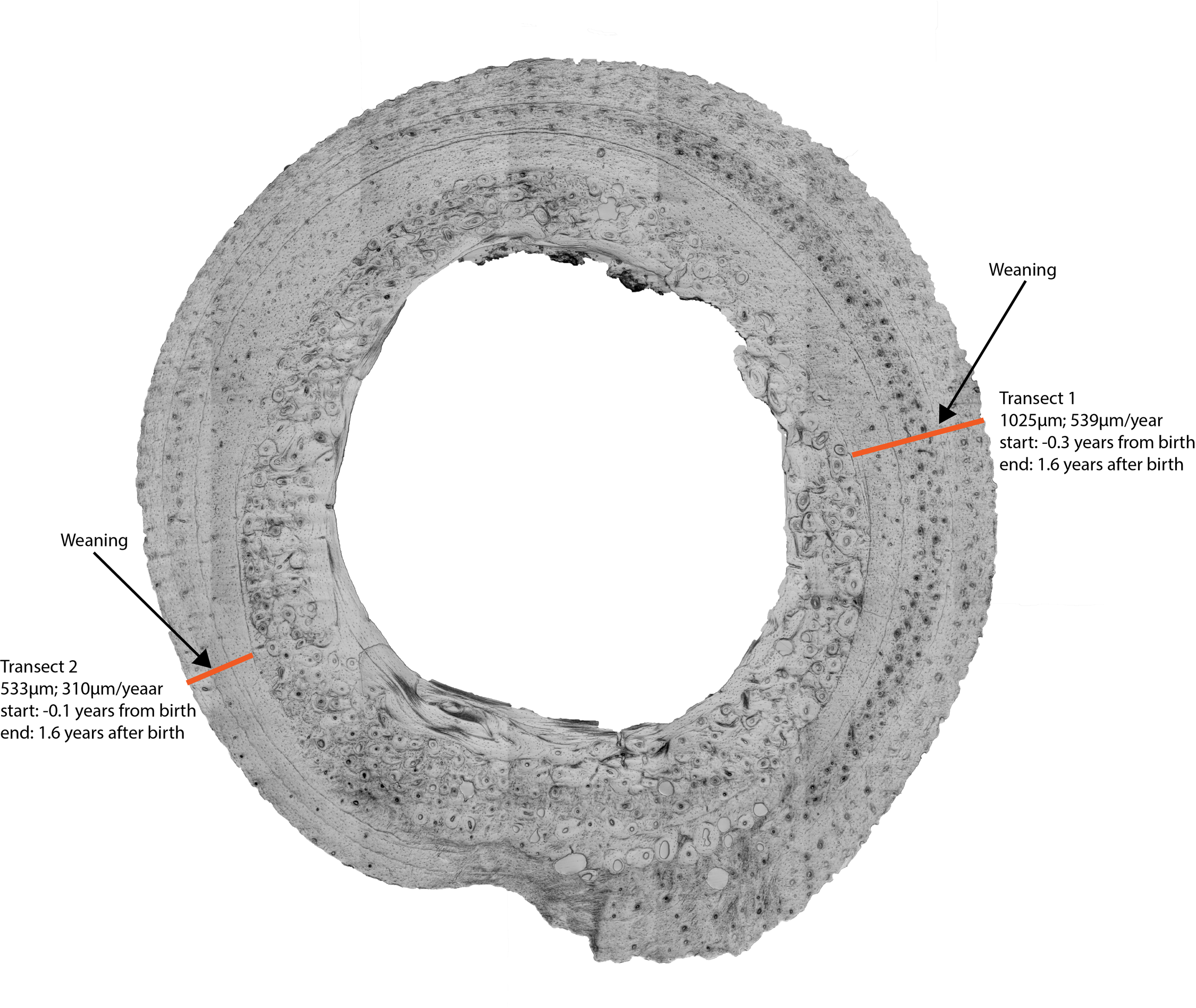

Supplement: S1 Fig — The red lines indicate the locations of transects 1 and 2. For each transect we report the length in micrometers; the yearly growth rate; the age range covered by the transect. For a description of the method used to derive the ages, see the Methods section of the main manuscript. (TIF) [file pone.0276866.s001.tif]

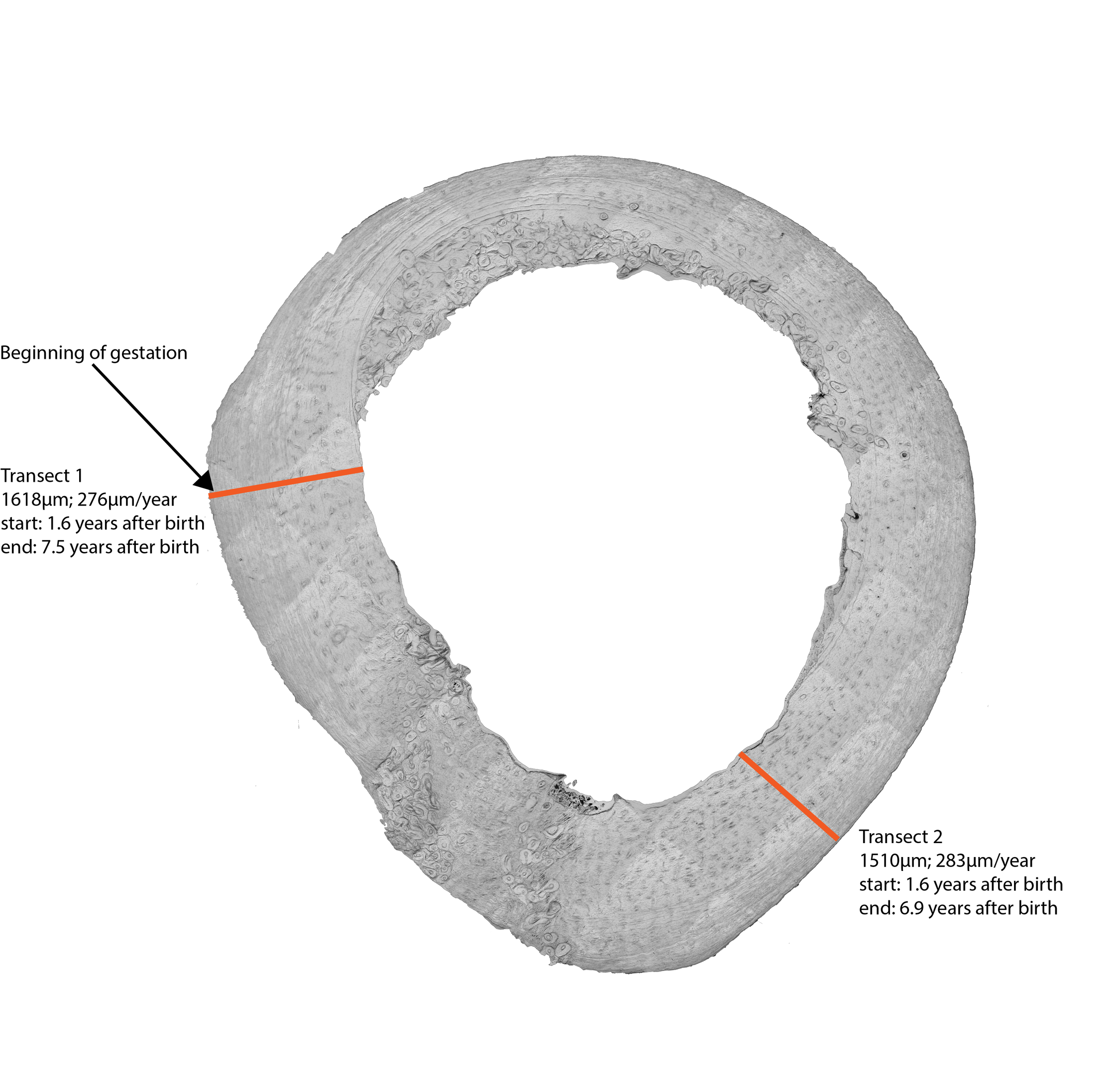

Supplement: S2 Fig — The red lines indicate the locations of transects 1 and 2. For each transect we report the length in micrometers; the yearly growth rate; the age range covered by the transect. For a description of the method used to derive the ages, see the Methods section of the main manuscript. (TIF) [file pone.0276866.s002.tif]

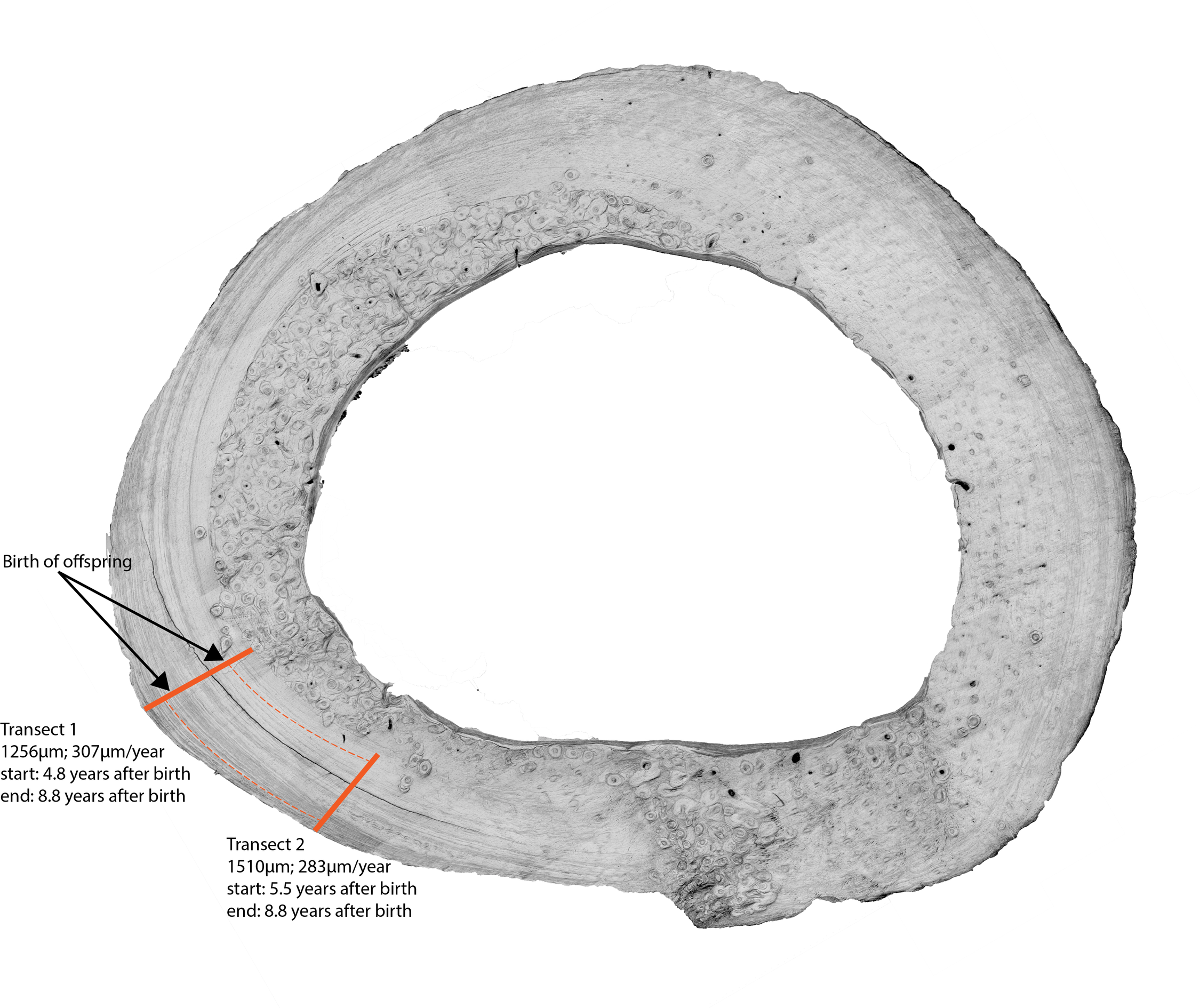

Supplement: S3 Fig — Montaged micrograph of the femur of M526 obtained in reflected light. The red lines indicate the locations of transects 1 and 2. For each transect we report the length in micrometers; the yearly growth rate; the age range covered by the transect. For a description of the method used to derive the ages, see the Methods section of the main manuscript. (TIF) [file pone.0276866.s003.tif]

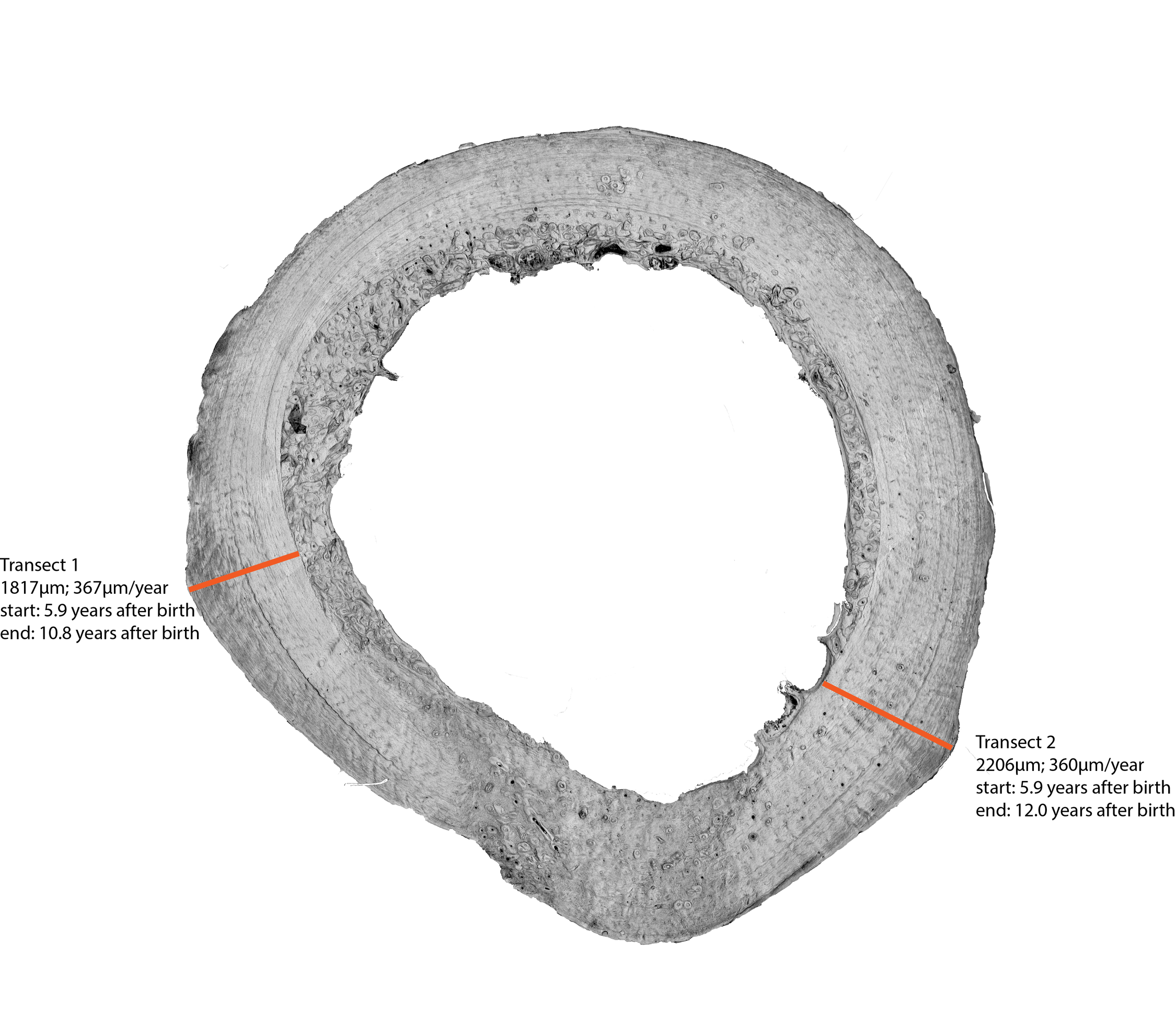

Supplement: S4 Fig — The red lines indicate the locations of transects 1 and 2. For each transect we report the length in micrometers; the yearly growth rate; the age range covered by the transect. For a description of the method used to derive the ages, see the Methods section of the main manuscript. (TIF) [file pone.0276866.s004.tif]

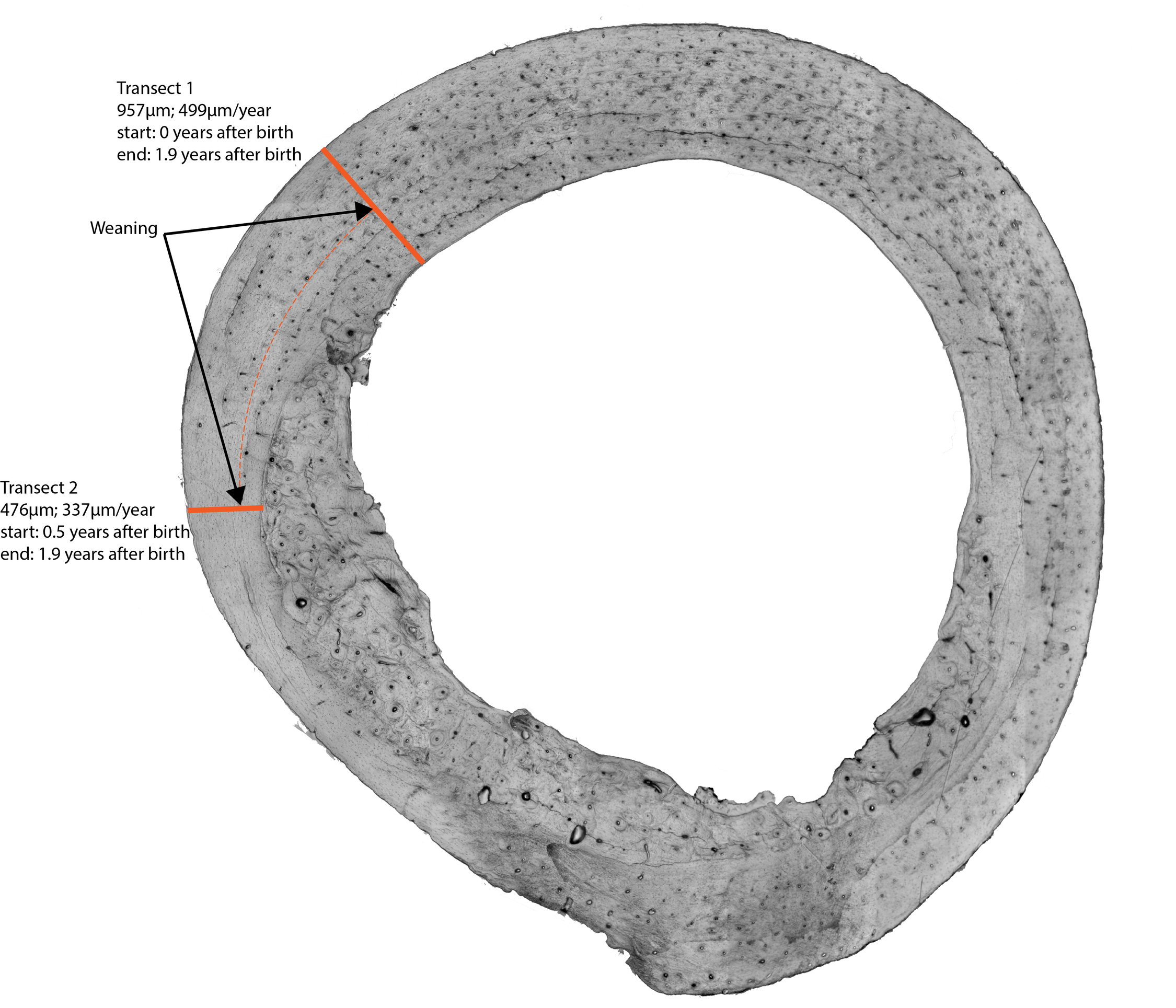

Supplement: S5 Fig — The red lines indicate the locations of transects 1 and 2. For each transect we report the length in micrometers; the yearly growth rate; the age range covered by the transect. For a description of the method used to derive the ages, see the Methods section of the main manuscript. (TIF) [file pone.0276866.s005.tif]

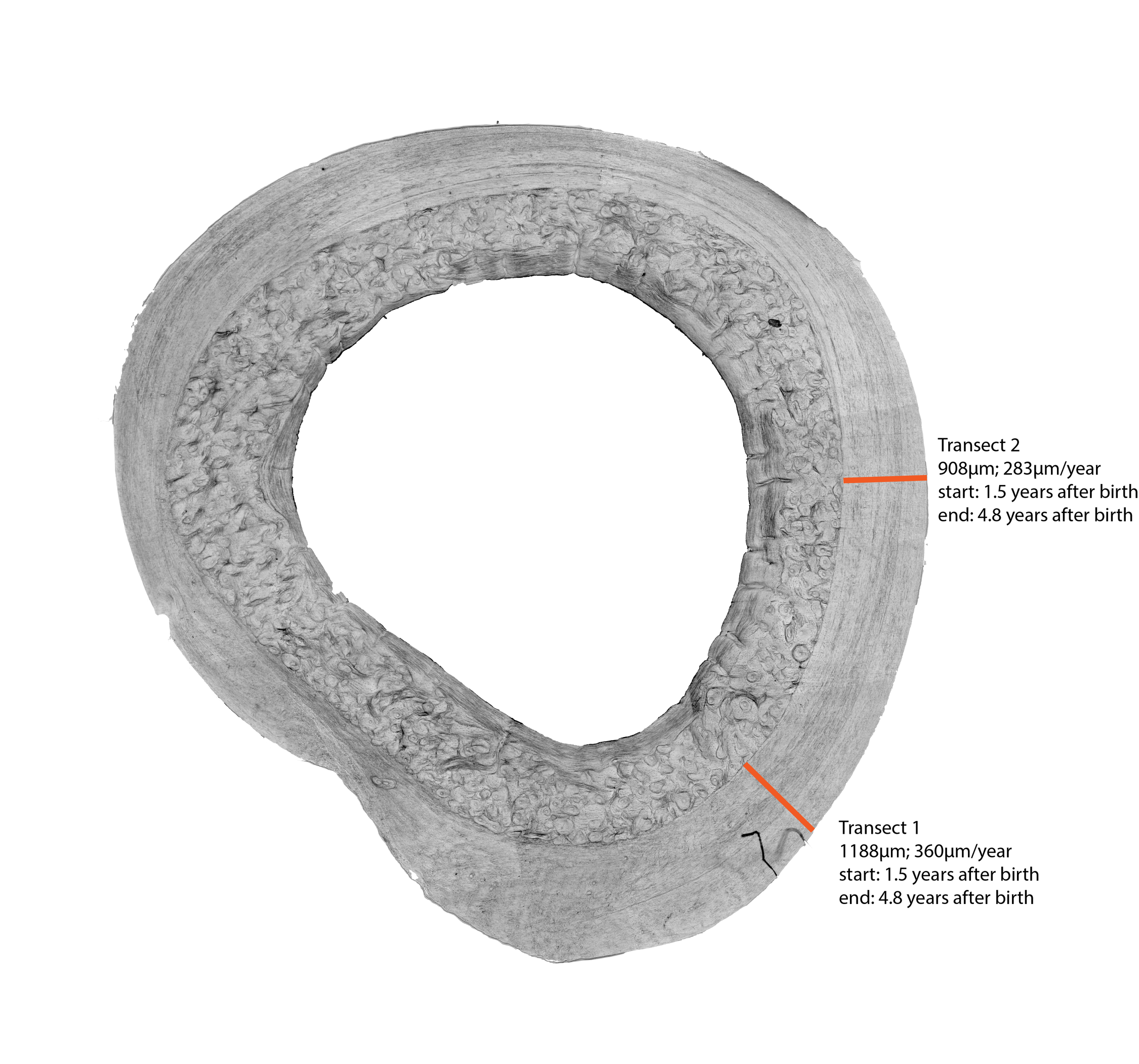

Supplement: S6 Fig — The red lines indicate the locations of transects 1 and 2. For each transect we report the length in micrometers; the yearly growth rate; the age range covered by the transect. For a description of the method used to derive the ages, see the Methods section of the main manuscript. (TIF) [file pone.0276866.s006.tif]

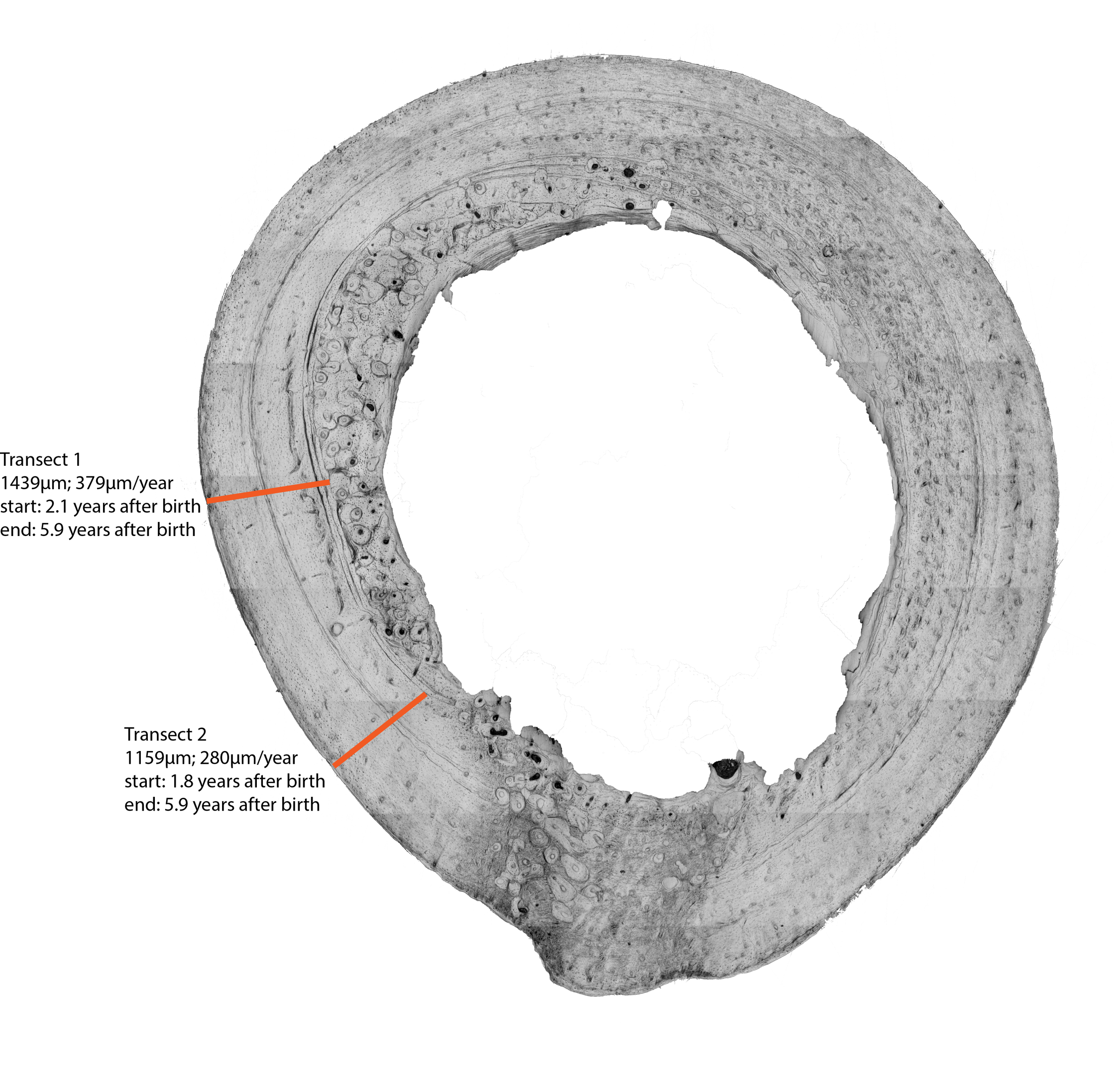

Supplement: S7 Fig — For each transect we report the length in micrometers; the yearly growth rate; the age range covered by the transect. For a description of the method used to derive the ages, see the Methods section of the main manuscript. (TIF) [file pone.0276866.s007.tif]

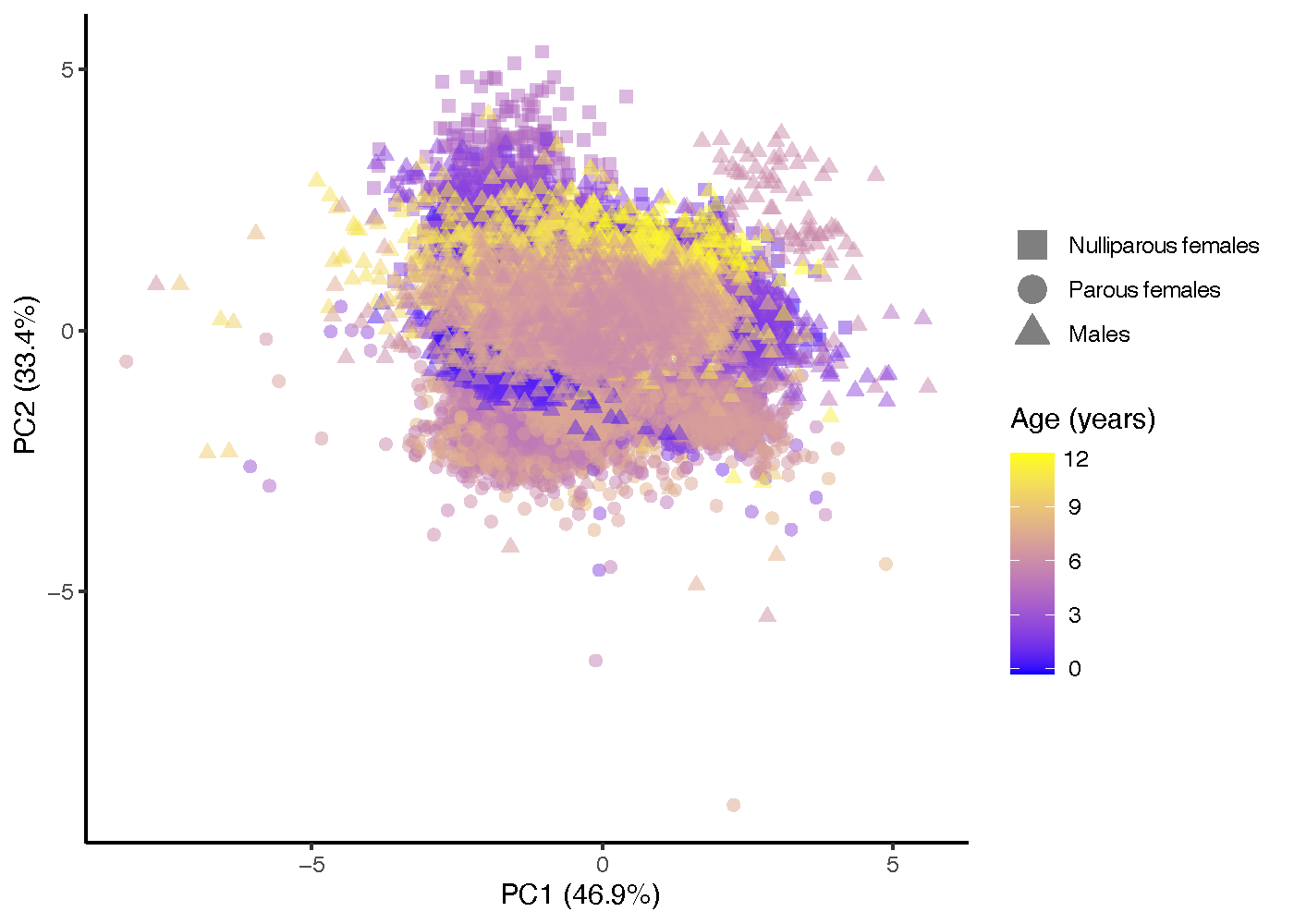

Supplement: S8 Fig — As visible by the superimposition between blue and yellow datapoints, age is not a discriminating factor. (TIF) [file pone.0276866.s008.tif]

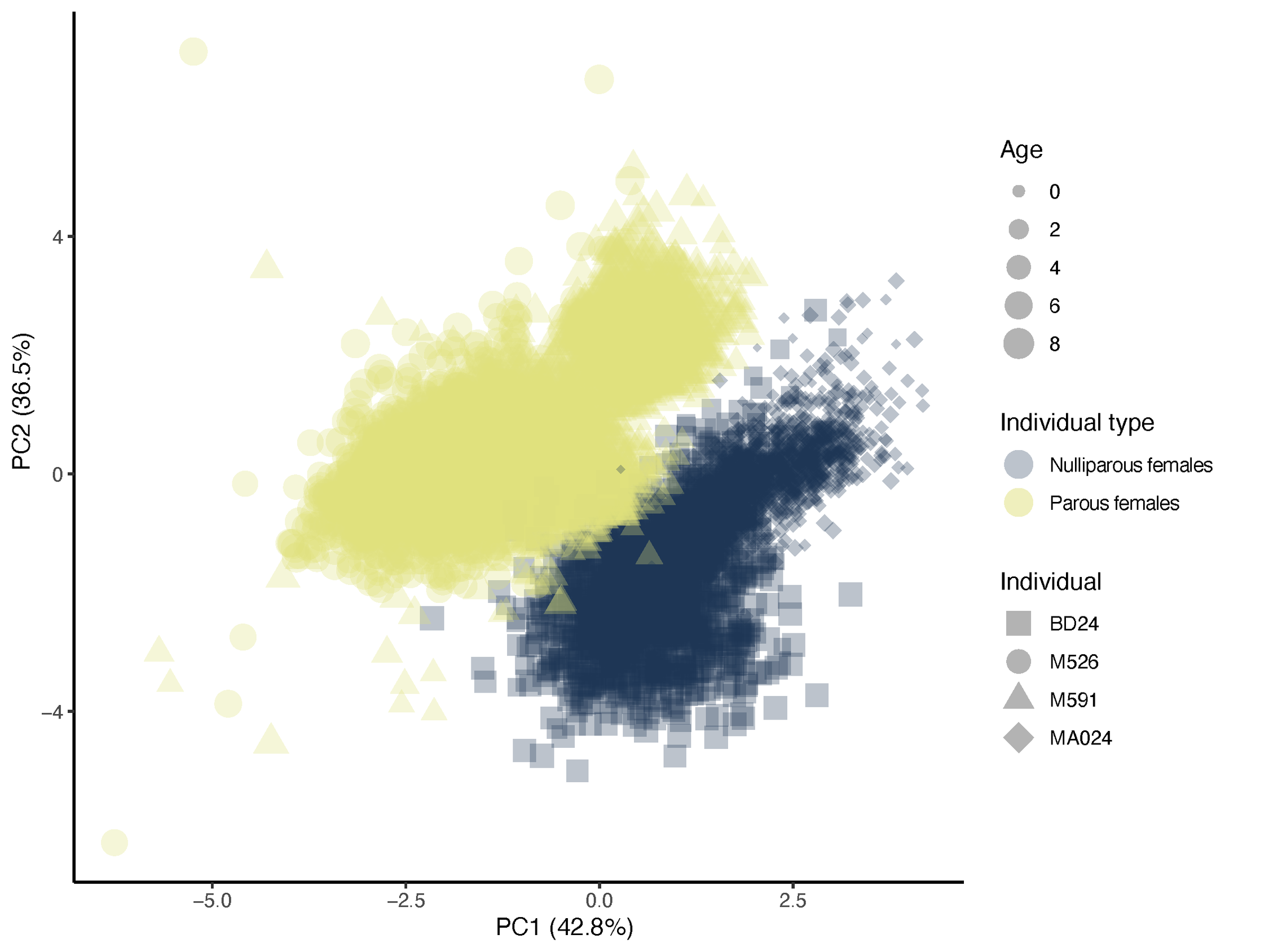

Supplement: S9 Fig — The data from both segments of the same individual are pooled together. There is clear separation between parous and nulliparous females. Dot size is proportional to age. It is clearly visible that the separation between parous and nulliparous females is independent of age (dot size). (TIF) [file pone.0276866.s009.tif]

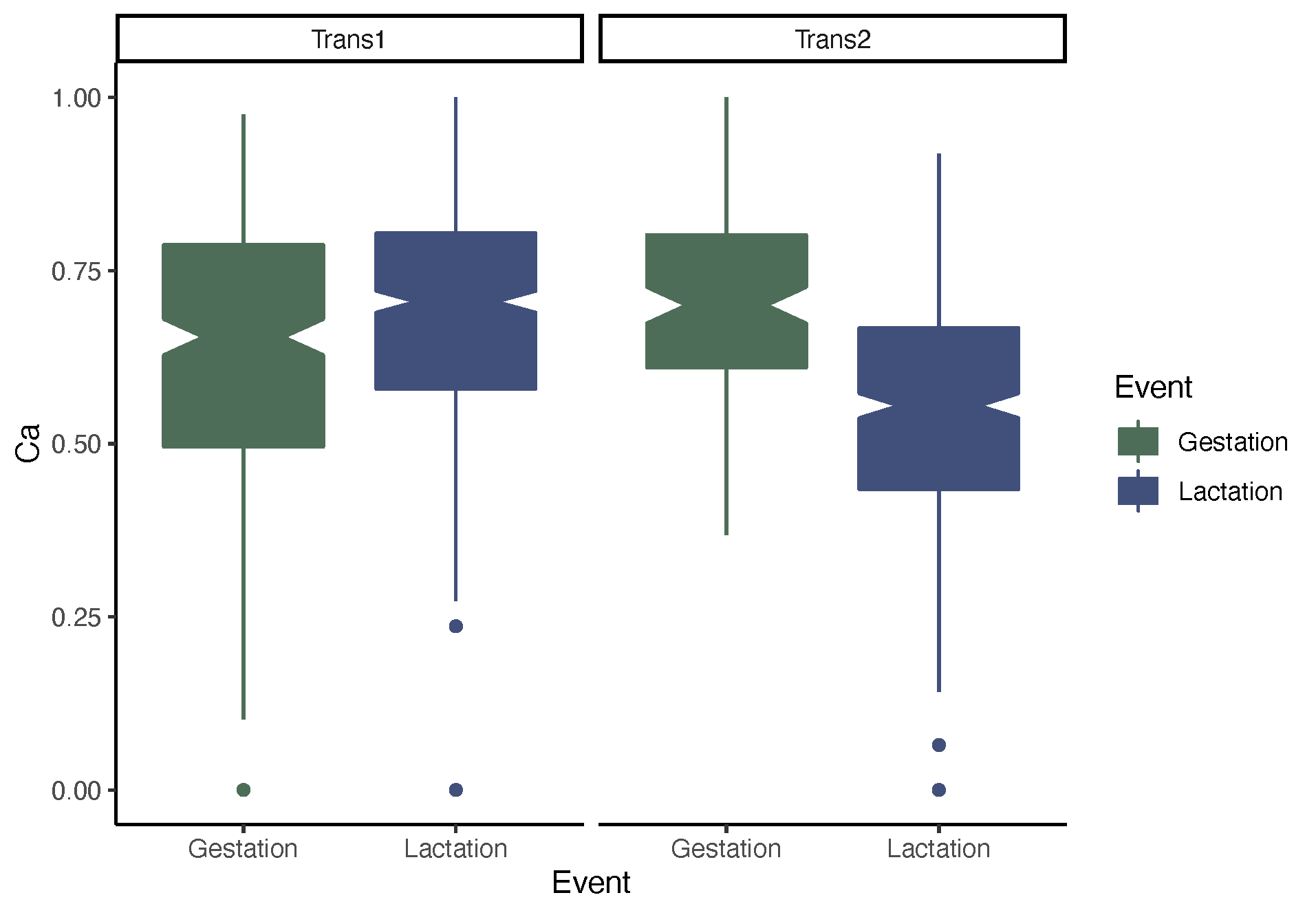

Supplement: S10 Fig — Ca values are significantly lower only in Transect 2 (p<0.00001). (TIF) [file pone.0276866.s010.tif]

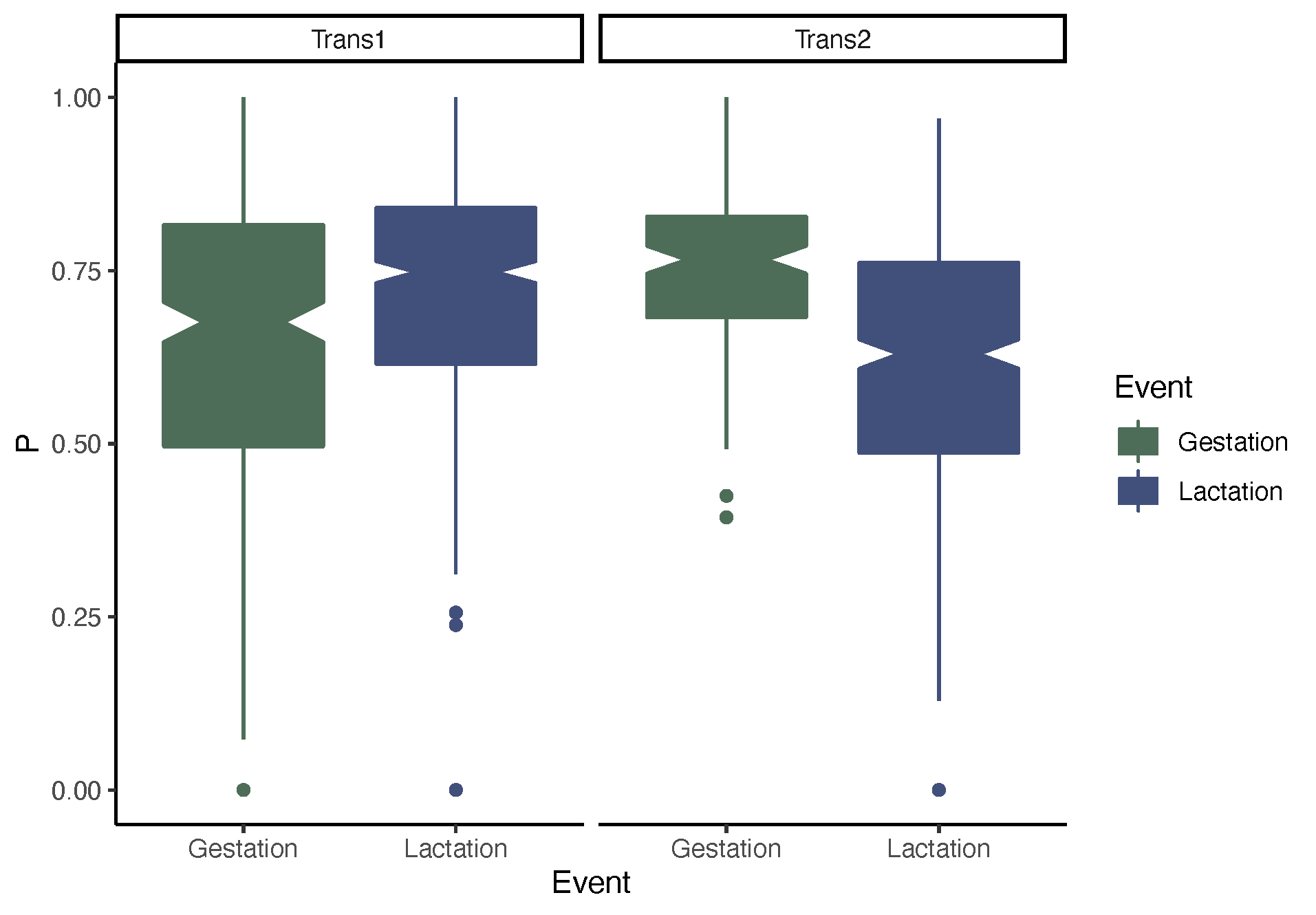

Supplement: S11 Fig — P values are significantly lower only in Transect 2 (p<0.00001). (TIF) [file pone.0276866.s011.tif]

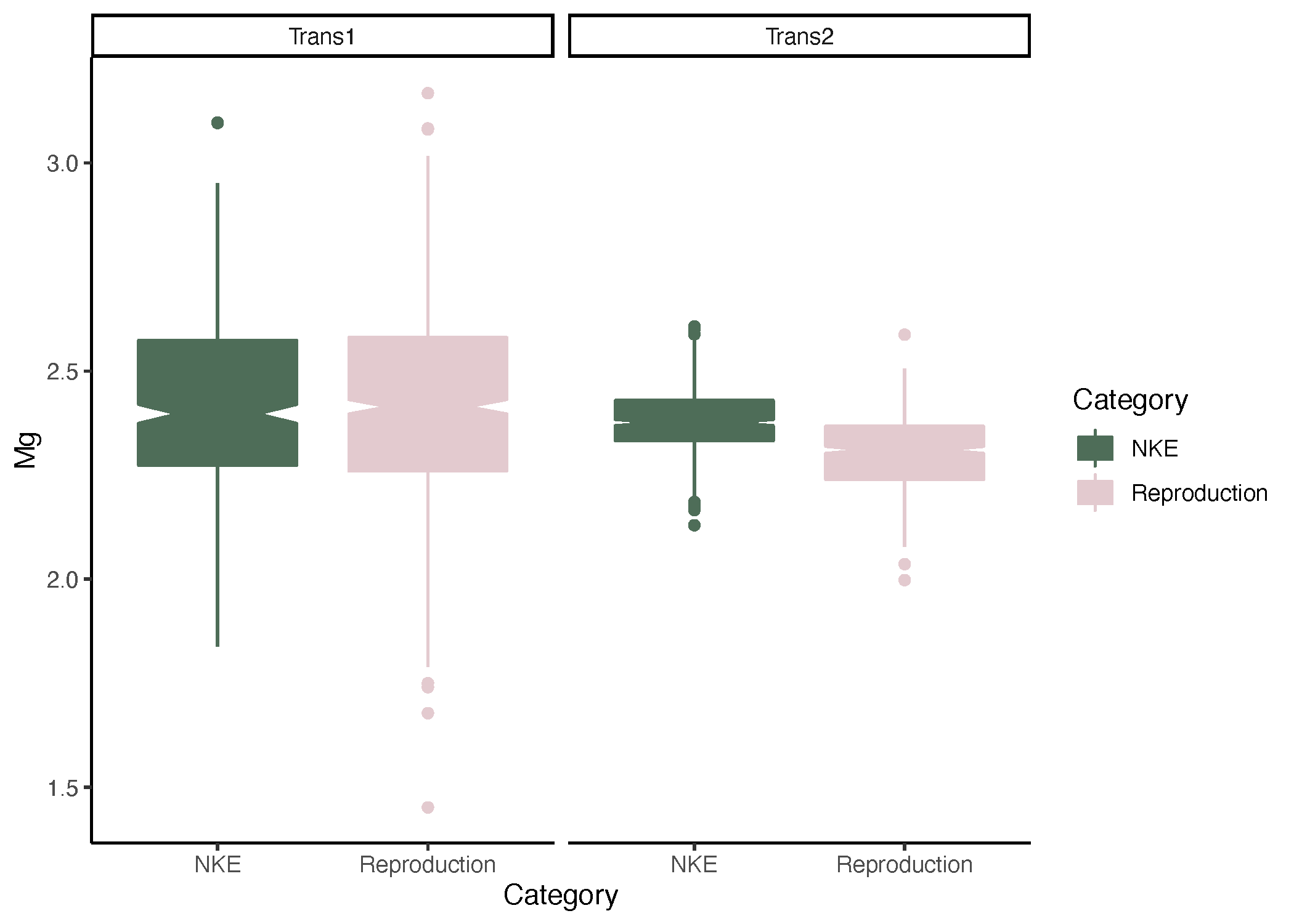

Supplement: S12 Fig — There is a significant difference only in Transect 2 (p<0.001). (TIF) [file pone.0276866.s012.tif]

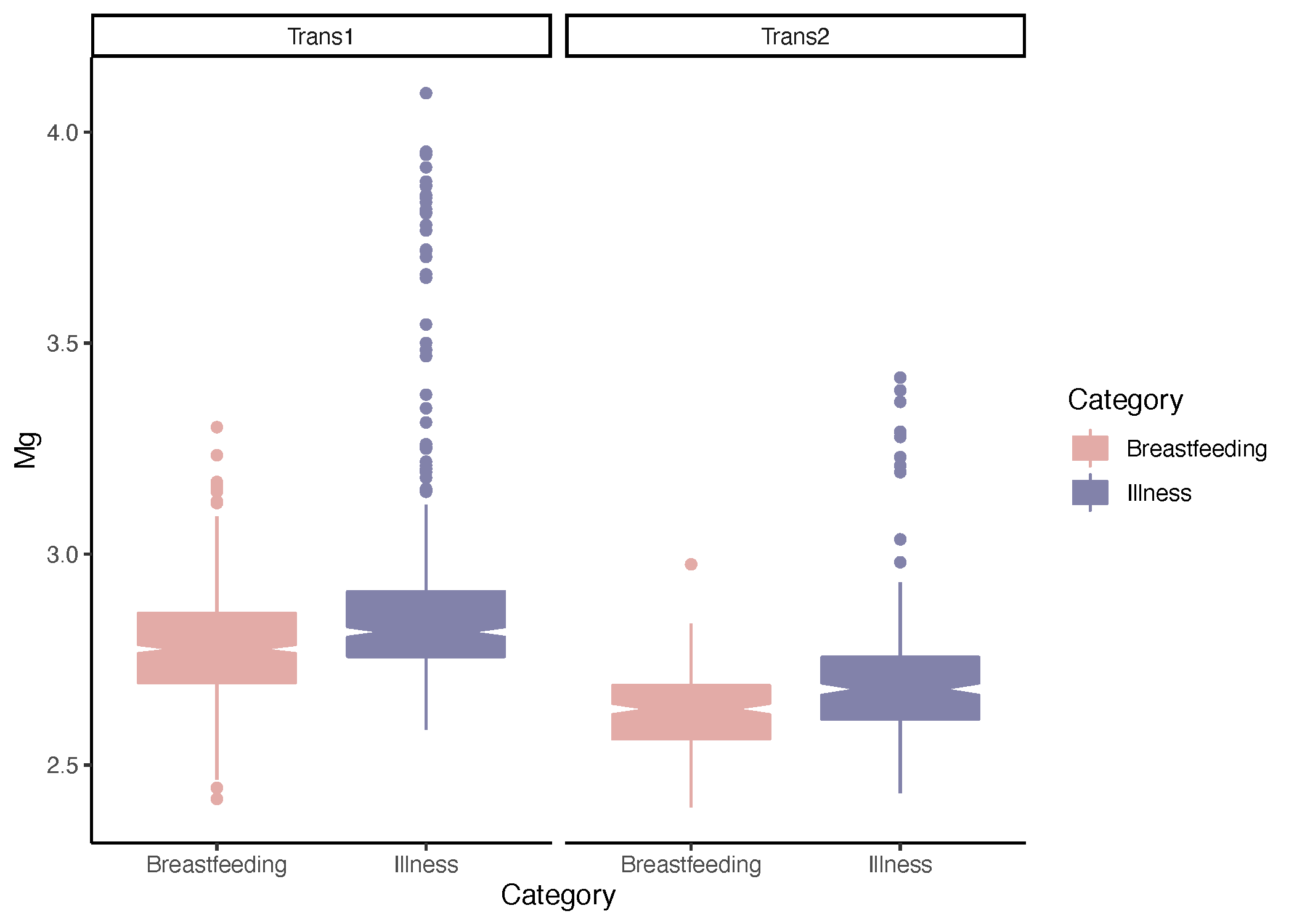

Supplement: S13 Fig — The difference is significant in both transect 1 and transect 2 (p<0.00001). (TIF) [file pone.0276866.s013.tif]

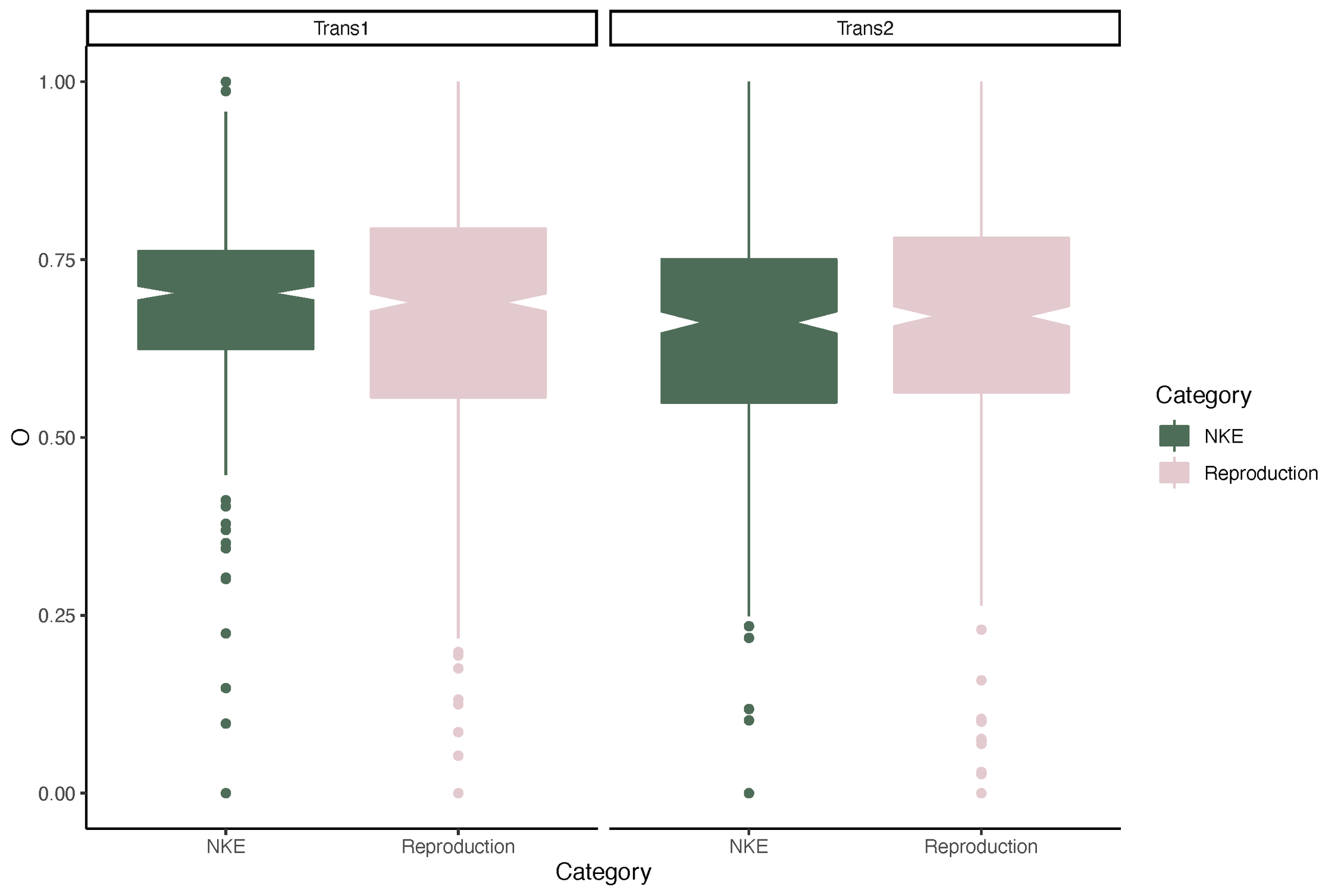

Supplement: S14 Fig — (TIF) [file pone.0276866.s014.tif]
