# Supplementary material for: Elemental composition of primary lamellar bone differs between parous and nulliparous rhesus macaque females
Source: PLoS One. 2022 Nov 1;17(11):e0276866. doi: 10.1371/journal.pone.0276866 (PMC9624403; doi:10.1371/journal.pone.0276866)
Supplement: S1 File — (DOCX) [file pone.0276866.s018.docx]

**S1 File. Supplementary methods and scripts.**

**Data analysis**

For each datafile we used the proprietary function *pair.test.all* to automatically run t-tests on all the five elements analyzed with individual type (male, parous female, nulliparous female) as categorical response variables. This function created an output file with the results of all the t-tests and “ns” if the test returned non-significant p-values. The function automatically adjusted the alpha threshold according to the Bonferroni correction, depending on the number of comparisons being made. Another function, *remove.ns*, removed the non-significant tests form the previous output. Finally, the function *final*, analyzed the outputs of *remove.ns* and coded each t-test as “take – yes” or “take – no” depending on whether for each element the number of significant t-tests was equal (take – yes) or inferior (take – no) to the total number of t-tests. This was done to identify those elements that not only were significantly different between two variables of a category but were also significant between all variables of a category. For example, Ca would be taken to univocally identify parturitions (take – yes) only if the difference in Ca values was significant between one type of individual and all other ones, but not if Ca was significantly different between parous females and male, but not between parous females and nulliparous ones. The code for all custom functions is available in the in the present document, in the section Supplementary Scripts.

**Scripts**

1. **pair.test.all**

ptest = function(dta, col){

dta %>%

rstatix::pairwise_t_test(as.formula(paste0(col," ~ Season"))) **#<==== change variable here**

}

pair.test.all = function (data, a=11, b=ncol(data)) { **#<==== change the value of a accordingly here**

y=NULL

for (i in colnames(data[a:b])) {

results = ptest(data, i)

y=rbind(y, results)

#print(data.frame(results))

}

y = y %>% add_column(n_comps = NA)

ncomps = length(unique(data$Season)) -1 **#<==== change variable here**

y$n_comps[is.na(y$n_comps)] = ncomps

y<<- data.frame(y)

write.csv(y,file=paste0("results_", deparse(substitute(data))), row.names=FALSE)

assign(x = paste0("results_", deparse(substitute(data))),

value =y, envir = .GlobalEnv)

rm(y, envir = .GlobalEnv)

rm(list = as.character(substitute(data)), envir = .GlobalEnv)

}

*NOTE: to work, the variables with the elements must be sequential to each other and be the last ones of the dataset. “a” must be adjusted manually to correspond to the first column containing elemental data*. *If these conditions are not satisfied the function will return an error. This function saves an output file in the working directory with the results of the t-tests. The output file will be called “results_data”, that is: results is added to the original name of the file.*

1. **remove.ns**

remove.ns = function(my.data) {

new.data = my.data[!my.data$p.adj.signif=="ns", ]

y <<- data.frame(new.data)

write.csv(y,file=paste0("sig_", deparse(substitute(my.data))), row.names=FALSE)

assign(x = paste0("sig_", deparse(substitute(my.data))),

value =y, envir = .GlobalEnv)

rm(y, envir = .GlobalEnv)

rm(list = as.character(substitute(my.data)), envir = .GlobalEnv)

}

*Note: this function will delete the input file from the global environment and save an output file in the working directory with only the t-tests that were significant. The output file will be called “sig_results_data”, that is: sig_results is added to the original name of the file.*

1. **final**

final = function(my.data) {

y = my.data %>% add_column(take = NA)

new.data = y %>% gather(key = "group", value = "category", group1, group2)

new.data = new.data %>% group_by(.y.) %>% add_count(category)

new.data<<- new.data

ncomp = my.data$n_comps[1]

for (i in 1:nrow(new.data)) {

if (new.data$n[i] == ncomp) {

new.data$take[i] = "yes"

}

else {

new.data$take[i] = "no"

}

}

new.data <<- data.frame(new.data)

write.csv(new.data, file = paste0("ok_", deparse(substitute(my.data))), row.names=FALSE)

assign(x = deparse(substitute(my.data)),

value = new.data, envir = .GlobalEnv)

rm(new.data, envir = .GlobalEnv)

}

*Note: this function will save an output file in the working directory with only the t-tests that were significant and an additional column indicating whether that element is significantly different between all the comparisons made. The new variable will be called “take” and the values it can assume are either “yes” or “no”. The output file will be called “ok_sig_results_data”, that is: ok_sig_results is added to the original name of the file.*
